# Health literacy and self-care in patients with heart failure: a cross-sectional study

**DOI:** 10.3389/fpubh.2025.1657807

**Published:** 2026-01-21

**Authors:** Azucena Santillan-Garcia, Vicente Gea-Caballero, Elisa Frutos-Bernal, Raúl Juárez-Vela, Antonio Martínez-Sabater, Enrique Castro-Sánchez, ALFABETIC Collaborative Working Group, Ana Cristina Cabellos-Garcia

**Affiliations:** 1 Cardiology Department, University Hospital of Burgos, Burgos, Spain; 2 Faculty of Health Science, Research Group Community Health and Care SALCOM, Valencian International University, Valencia, Spain; 3 Faculty of Medicine, University of Salamanca, Salamanca, Spain; 4 Faculty of Health Sciences, Department of Nursing, Research Group in Care GRUPAC, University of La Rioja, Logroño, Spain; 5 Nursing Department, Nursing Care and Education Research Group (GRIECE), University of Valencia, Valencia, Spain; 6 Care Research Group (INCLIVA), Hospital Clínico Universitario of Valencia, Valencia, Spain; 7 Health Protection Research Unit in Healthcare-Associated Infection and Antimicrobial Resistance at Imperial College London, National Institute for Health Research, London, United Kingdom

**Keywords:** determinants of health, health inequality, health literacy, health outcomes, heart failure, self-care

## Abstract

**Background:**

Health literacy (HL) is recognized as a key determinant in the management of chronic diseases, including heart failure (HF). Adequate HL facilitates understanding and application of health information, promoting effective self-care and improved clinical outcomes. In contrast, low HL is associated with poorer disease control, higher hospitalization rates, and increased mortality. Despite its importance, the relationship between HL and self-care in HF patients remains underexplored in specific sociodemographic and clinical contexts.

**Objective:**

This study aimed to assess HL and self-care capacity in individuals with HF, identify associated sociodemographic and clinical variables, and explore the predictive value of HL on self-care behaviors.

**Methods:**

A cross-sectional, observational study was conducted among 195 HF patients attending a referral center in Burgos, Spain. HL was assessed using the European health literacy survey questionnaire (HLS-EU-Q16), while self-care was evaluated using the European heart failure self-care behavior scale (EHFScBS). Data were collected via telephone interviews. Descriptive and inferential statistics were applied, including correlation and multiple linear regression analyzes.

**Results:**

The mean age of participants was 69.26 (±9 years), with a predominance of males (83.1%). Most participants reported medium social class (87.7%) and basic education (64.6%). HL levels were significantly associated with educational attainment, social class, and gender; women and individuals with higher education levels demonstrated greater HL scores. Regression analysis revealed that HL negatively predicted self-care scores (*p* < 0.001), as higher EHFScB-9 scores indicate poorer self-care, and in our study higher HL was associated with lower EHFScB-9 scores (*r* = −0.320; *β* = −0.189), which is consistent with better self-care behaviors. Older age and lower educational attainment were linked to lower HL and poorer self-care. Notably, female sex and upper-middle social class were also significant predictors of self-care capacity.

**Conclusion:**

Health literacy is a significant predictor of self-care in HF patients, and its levels are influenced by key sociodemographic variables. These findings underscore the necessity of incorporating HL assessment into clinical practice and tailoring educational interventions to address disparities. Enhancing HL could promote more effective self-management and potentially reduce adverse outcomes in HF populations. Future research should focus on longitudinal analyzes and the development of targeted, equitable interventions based on HL profiles.

## 1 Introduction

Health literacy (HL) refers to the individual (skills, abilities, and knowledge) and social factors that influence the acquisition, processing, and understanding of information related to health and health-related systems in order to make appropriate decisions for the well-being of the individual (1). Additionally, HL includes the ability to read and understand health instructions, or follow treatment plans. HL is determinant in the prevention and management of chronic diseases such as diabetes, chronic kidney disease, and chronic obstructive pulmonary disease (COPD), and it is closely linked social determinants fuelling social and health inequalities (2, 3). Patients with low HL have difficulties with therapeutic concordance and effective disease management, resulting in worse health outcomes, higher hospitalization rates, and lower quality of life (4, 5).

Although HL is a determinant of health protection and disease prevention, it is in itself also influenced by a wide range of personal, social, and structural variables (6–8). Among these factors, for example, schooling, socioeconomic status, and gender-albeit from an intersectional lens and as a reflection of inequalities in access to education (7, 9, 10)-as well as urbanicity (11) have been described. Measuring HL is essential to identify inequalities in access, understanding and use of health information, which allows for the design of more effective and equitable interventions. There are multiple tools developed in recent decades, such as the rapid estimate of adult literacy in medicine (REALM), the test of functional health literacy in adults (TOFHLA) and the newest vital sign (NVS), which have been widely used, although they have limitations by focusing primarily on functional literacy and not addressing broader dimensions such as critical or communicative literacy (12). More recent instruments, such as the health literacy questionnaire (HLQ) and the European health literacy survey questionnaire (HLS-EU-Q47), offer a more comprehensive assessment by considering multiple dimensions of health literacy (13). However, the lack of consensus on the dimensions assessed and the variability in the psychometric properties of these tools indicate the need to develop more robust instruments adapted to diverse population contexts. Accurate measurement of HL is essential to improve health outcomes and reduce existing inequalities (14).

Heart failure (HF) is a chronic and progressive health problem affecting more than 64 million people worldwide (49). Optimal management is multimodal, including pharmacological therapies, assistance and continuous healthcare, and high patient involvement in self-management (15). HL is therefore equally crucial in the management of HF; people with HF and low HL have worse health outcomes, including higher rate of re-hospitalizations and mortality (16, 17), as well as higher frailty burden (18), worse self-care skills (19) and reduced quality of life (20). According to a study by Smith et al. (21), greater understanding of medical information reduces these cardiovascular risks. According to Cabellos-García et al. (22), patients with low literacy have 30% higher cardiovascular risk and increased undesirable health outcomes. For example, inadequate management of arterial hypertension, poor cholesterol control or problems following a certain diet, all of which are fundamental risk factors in the control of cardiovascular problems (49).

Consequently, appropriate lifestyles and capacity for the management, control and evaluation of HF are key, and are modulated by the level of HL. This can be done by patients themselves, but also by their caregivers, as it is very common for patients to need partial or total support from a caregiver at advanced stages of the disease. In these cases, the caregiver plays a substitute role that includes decisions about the patient’s diet or activity, pharmacological treatment and management of appointments with professionals, or decisions such as whether or not to go to the emergency room. Regardless of who performs it, it is what we know as self-care (23), a key concept that can be known since it can be measured, for example with the European heart failure self-care behavior scale (EHFSCBS), a scale that measures self-care capacity in a brief form in both patients and caregivers (24).

HL competencies associated with communication and socialization are critical. In general, there is a positive association between self-care and social support, key factors for effective HF management (18), and people with higher HL have greater needs for communication about their disease and self-care (25). Finally, the HL level of caregivers also influences the self-care of people with HF (26). This information can inform us about patients or caregivers who may have compromised their ability and skills to live with their disease, their self-care, and their capacity for correct decision making. Knowing this information would allow us to implement actions aimed at improving these skills and controlling the risk of undesirable events.

To improve HL in people living with HF, various interventions have been developed. These include assessing HL using validated questionnaires and its impact on therapeutic adherence (27), personalized education (28), care models based on nurses supported by digital platforms, and programs aimed at improving the HL of caregivers (26). It is therefore necessary to conduct research that analyzes the level of health literacy and its relationship with quality of life in this population, in order to design more effective and personalized educational interventions and care strategies.

Thus, we set out to determine the level of HL and self-care in a sample of people with HF, and to describe the relationship between this level of self-care and clinical and sociodemographic variables routinely collected during social and health care in a referral center. Secondarily, we intend to explore the predictive validity of the level of HL (measured with HLS-EU-Q16) on the self-care capacity of people with HF and their caregivers.

## 2 Materials and methods

### 2.1 Design

An observational, cross-sectional study was conducted in people with HF accessing a referral service in Burgos (Spain) in 2023.

### 2.2 Study population

Persons attending the consultation of the heart failure unit of the Hospital Universitario de Burgos (Spain) during the study period (in July 2022 *N* = 321), who voluntarily agreed to participate and who could be reached by telephone. After eliminating those who refused to participate and could not be reached, the initial sample is *n* = 264.

As inclusion criteria, we focused on people between 50 and 85 years of age with a previous diagnosis of HF and with established pharmacological treatment. Exclusion criteria included sensory impairments that prevented completion of the questionnaires used, illiteracy, severe neurocognitive or mental health problems that prevented the patient from understanding their pathology or vitiated their consent to participate in the study.

### 2.3 Variables

Different sociodemographic variables were collected (place of residence, perceived social class, sex, and educational level), clinical variables (etiology of HF, obesity, ejection fraction, functional stage, among others) and the completion of the health literacy survey—European Union questionnaires in Spanish (HLS-EU-Q16) (29) and the Spanish version of the European heart failure self-care scale (30) [European heart failure self-care behavior scale (EHFScBS)].

The EHFScBS, developed in 2003, assesses self-care in people with HF by means of 12 items with 5-point Likert-type responses (1 represents “completely agree/always,” and 5 “completely disagree/never”). The total score ranges from 12 (best self-care) to 60 (worst self-care).

The HLS-EU-Q16 (abbreviated version of the HLS-EU-Q47 questionnaire developed to measure HL among the general population of the European Union, focuses on three fundamental areas: health care, disease prevention and health promotion). The short version validated in Spain consists of 16 items with 4-point Likert-type responses (very difficult, difficult, easy, easy, very easy), which allows a quick and efficient assessment of HL. According to the score obtained, HL is classified into three levels: inadequate (0–8 points), problematic (9–12 points), or adequate (13–16 points). This instrument can be self-administered or interviewed, and is useful for identifying barriers in the handling of health information and designing adapted educational interventions. It showed a Cronbach’s alpha of 0.92 in a unidimensional structure.

All variables except for the EHFScBC and the HLS-EU-Q16 are recorded in the health system’s own database. The EHFScBC and HLS-EU-Q16 questionnaires were completed by patients via telephone interview conducted by trained expert nurses between the months of February and April 2023. Surveyed via telephone and recorded by an outside caller in a Microsoft Excel spreadsheet and subsequently exported for statistical analysis using IBM SPSS Statistics software.

### 2.4 Statistical analysis

Descriptive statistical analysis was performed for all study variables. Quantitative variables were measured using measures of central tendency (mean and median) and dispersion (standard deviation, interquartile range). Qualitative variables are presented in absolute and relative frequencies (percentages). To assess the relationship between the level of self-care (continuous variable) and sociodemographic variables, Student’s *t*-test or ANOVA (as appropriate to the data) was used to compare self-care means between different categories (e.g., according to sex, social class or educational level), and Pearson’s or Spearman’s correlation (according to the normality of the data) to examine the relationship between age and self-care score. The predictive capacity of the HL in relation to self-care capacity was also measured by exploring the relationship between HLS-EU-Q16 and EHFScBS using the correlation coefficient. Subsequently, a linear regression was calculated to proceed to explain the relationship between the total score in EHFSCBS from the total score in HLSQ16. The normality of quantitative variables was assessed with the Kolmogorov–Smirnov or Shapiro–Wilk test. The level of statistical significance was set at *p* < 0.05.

### 2.5 Ethical aspects

Consent was obtained from the ethics committee of Áreas de Salud de Burgos y Soria (ref. CEIM 2809) and participants gave verbal consent for participation.

## 3 Results

Of 321 patients eligible to participate (266 males and 55 females), 35 patients were excluded due to age, 33 patients died, and 57 patients could not be located or did not wish to participate. Therefore, the final sample size was 195 patients (162 males and 33 females). The median age of the participants was 69.26 ± 9, mostly of middle social class (87.7%), and with basic education (64.6%) (Table 1).

**Table 1:** Sociodemographic characteristics of the sample.

Age	Mean ± SD	*n*	69.26 ± 9
			%
Gender	Male	162	83.1%
	Female	33	16.9%
Social class	Low	20	10.3%
	Medium	171	87.7%
	High	3	1.5%
Educational level	No education	34	17.4%
	Basic education	126	64.6%
	University education	33	16.9%

### 3.1 Results related to health literacy

To evaluate the relationship between social determinants and level of HL, age and HLS-Q16 scores were correlated (Spearman correlation coefficient). Most of these associations were not statistically significant, with low coefficients and *p* values above 0.05. However, significant negative correlations were identified between age and specific items: HLS-Q16_5 (*r* = −0.224; *p* = 0.002), HLS-Q16_8 (*r* = −0.271; *p* < 0.001), HLS-Q16_11 (*r* = −0.224; *p* = 0.002), HLS-Q16_12 (*r* = −0.231; *p* = 0.001) and HLS-Q16_15 (*r* = −0.179; *p* = 0.013) (Table 2).

**Table 2:** Relationship between social determinants and HL level.

	Age and literacy Spearman’s correlation analysis		Gender				Educational level		Social class	
	Correlation coefficient	Sig. (bilateral)	*U* de Mann–Whitney	W de Wilcoxon	*Z*	Sig. asin. (bilateral)	*H* de Kruskal–Wallis	Sig. asin.	*H* de Kruskal–Wallis	Sig. asin.
HLS-Q16_1: find information about treatments associated with diseases that interest you.	−0.1	0.166	2.423	15.626	−0.893	0.372	18.21	<0.001	4.253	0.119
HLS-Q16_2: find out where to get professional help when you are ill (e.g., doctor, pharmacist, or psychologist).	−0.021	0.771	2.484.5	15.687.5	−0.703	0.482	8.055	0.018	5.714	0.057
HLS-Q16_3: understanding what your doctor tells you	−0.006	0.933	2.428.5	15.469.5	−0.871	0.384	3.691	0.158	5.78	0.056
HLS-Q16_4: understand the doctor’s or pharmacist’s instructions on how to take prescribed medications.	−0.02	0.777	2.253	15.456	−1.609	0.108	3.499	0.174	8.72	0.013
HLS-Q16_5: assess when you may need a second opinion from another doctor	−0.224^**^	0.002	2.480	3.041	−0.675	0.5	27.634	<0.001	6.417	0.04
HLS-Q16_6: use the information provided by your doctor to make decisions about your illness.	−0.048	0.501	2.440	3.001	−0.841	0.4	7.559	0.023	13.31	0.001
HLS-Q16_7: follow the instructions given by your doctor or pharmacist.	0.058	0.422	2.137	15.340	−2.068	0.039	3.432	0.18	9.34	0.009
HLS-Q16_8: find information on how to deal with mental health issues such as stress or depression.	−0.271^**^	>0.001	2.429	15.632	−0.858	0.391	32.807	<0.001	5.984	0.05
HLS-Q16_9: understanding health warnings related to habits such as smoking, lack of physical exercise, or excessive alcohol consumption	−0.04	0.579	2.149.5	15.352.5	−1.954	0.051	5.191	0.075	2.132	0.344
HLS-Q16_10: understanding why you need to undergo early disease screening or medical checkups (e.g., mammograms, blood sugar tests, and blood pressure checks)	−0.05	0.491	2.385.5	15.588.5	−1.105	0.269	9.576	0.008	7.339	0.025
HLS-Q16_11: assess the reliability of information on health risks appearing in the media (e.g., television, the Internet, or other media outlets).	−0.224^**^	0.02	2098	15.139	−1.951	0.051	16.431	<0.001	7.986	0.018
HLS-Q16_12: deciding how to protect yourself from disease based on information provided by the media (e.g., newspapers, brochures, the Internet, or other sources of information)	−0.231^**^	0.001	1872	15.075	−2.787	0.005	13.909	<0.001	9.536	0.008
HLS-Q16_13: find activities that are good for your mental well-being (e.g., meditation, exercise, walking, Pilates, etc.).	−0.138	0.055	1773.5	14.976.5	−3.182	0.001	11.475	0.003	6.5	0.039
HLS-Q16_14: understand the health advice given by family and friends	0.079	0.272	2.460	15.663	−0.793	0.428	0.987	0.611	8.921	0.012
HLS-Q16_15: understand information provided by the media on how to improve your health (e.g., Internet, newspapers, magazines)	−0.179^*^	0.013	2012.5	15.215.5	−2.303	0.021	15.826	<0.001	11.15	0.004
HLS-Q16_16: assess which of your daily habits affect your health (e.g., habits related to alcohol consumption, eating habits, exercise, etc.).	−0.019	0.79	1821.5	15.024.5	−3.171	0.002	5.35	0.069	3.493	0.174

The ** is the statistical significance. There is statistical evidence of a negative (inverse) relationship between these variables.

**Figure 1:**
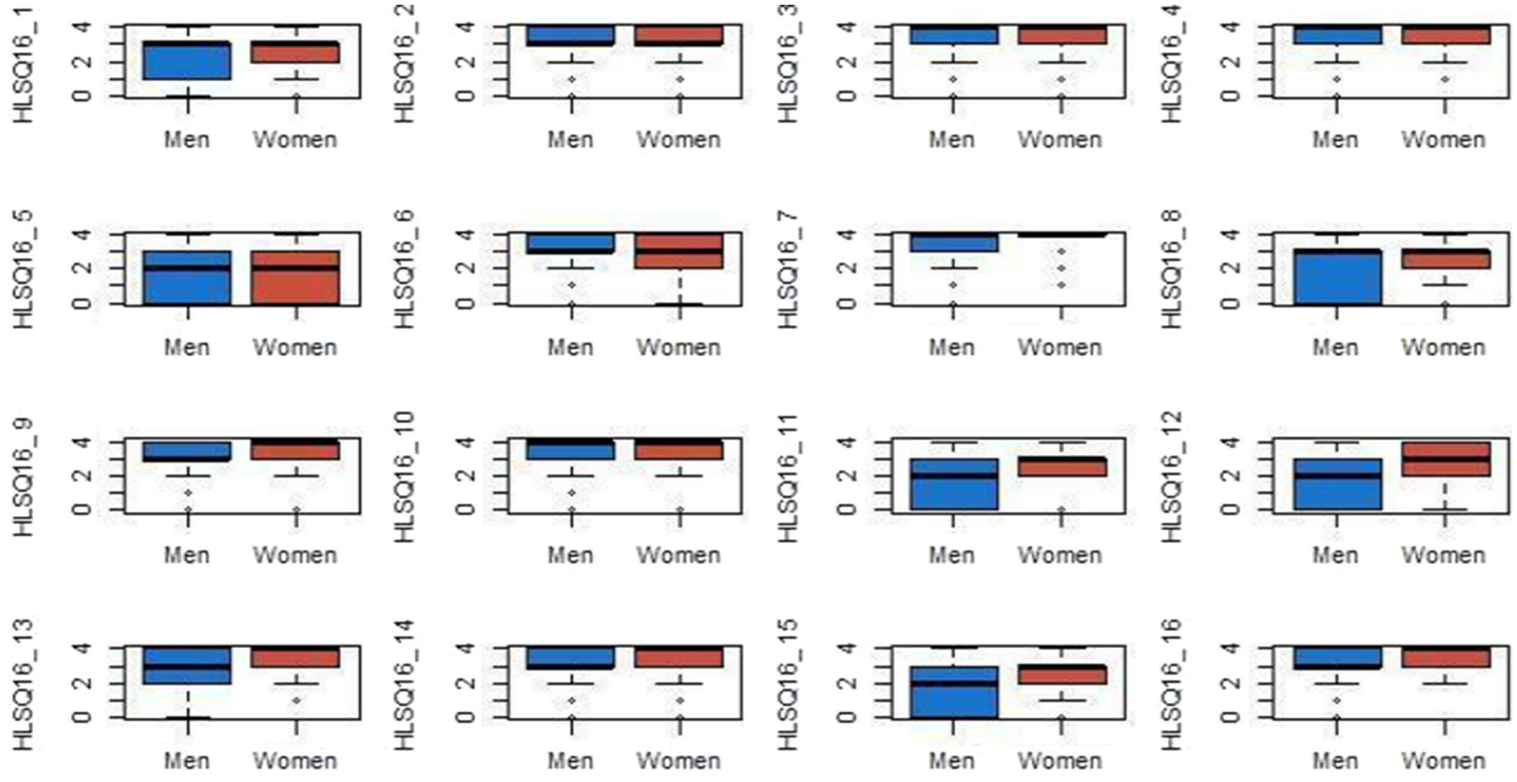
Gender and health literacy.

Gender analysis of HLS-Q16 scores using the Mann–Whitney *U* test again reveals significant differences in a minority of items—HLS-Q16_7 (*p* = 0.039), HLS-Q16_12 (*p* = 0.005), HLS-Q16_13 (*p* = 0.001), HLS-Q16_15 (*p* = 0.021), and HLS-Q16_16 (*p* = 0.002) (Figure 1). In all cases where significant differences were found, females obtained higher mean scores than males.

**Figure 2:**
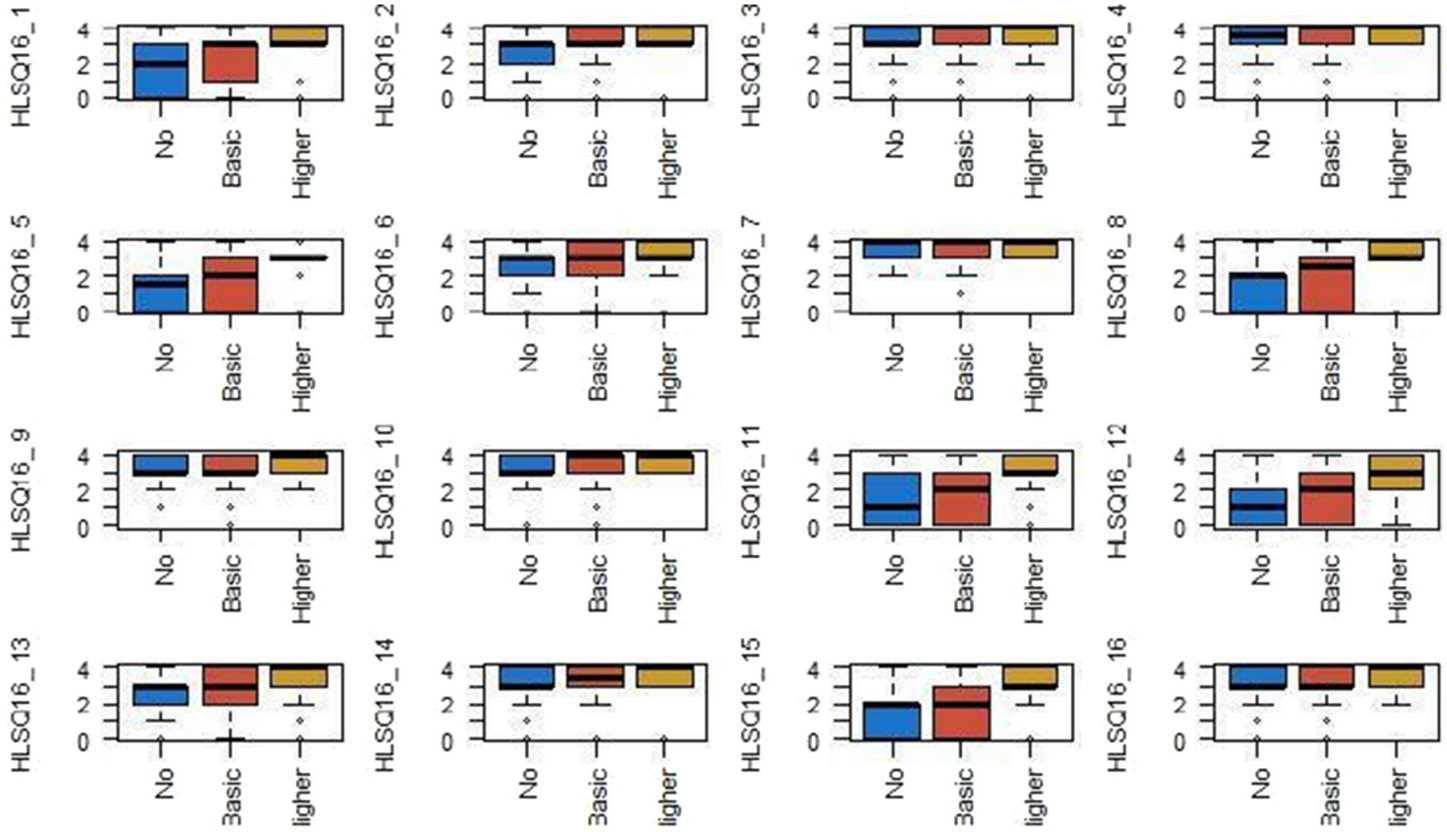
Educational level and health literacy.

Additionally, the results of the Kruskal-Wallis nonparametric analysis of variance show statistically significant differences in health literacy scores according to the educational level attained. The differences are evident in the items [HLS-Q16_1: HLS-Q16_2 (*p* < 0.001), HLS-Q16_2 (*p* = 0.018), HLS-Q16_5 (*p* < 0.001), HLS-Q16_8 (*p* < 0.001), HLS-Q16_10 (*p* = 0.008), HLS-Q16_11 (*p* < 0.001), HLS-Q16_12 (*p* < 0.001), HLS-Q16_13 (*p* = 0.003), HLS-Q16_15 (*p* < 0.001)] (Figure 2).

**Figure 3:**
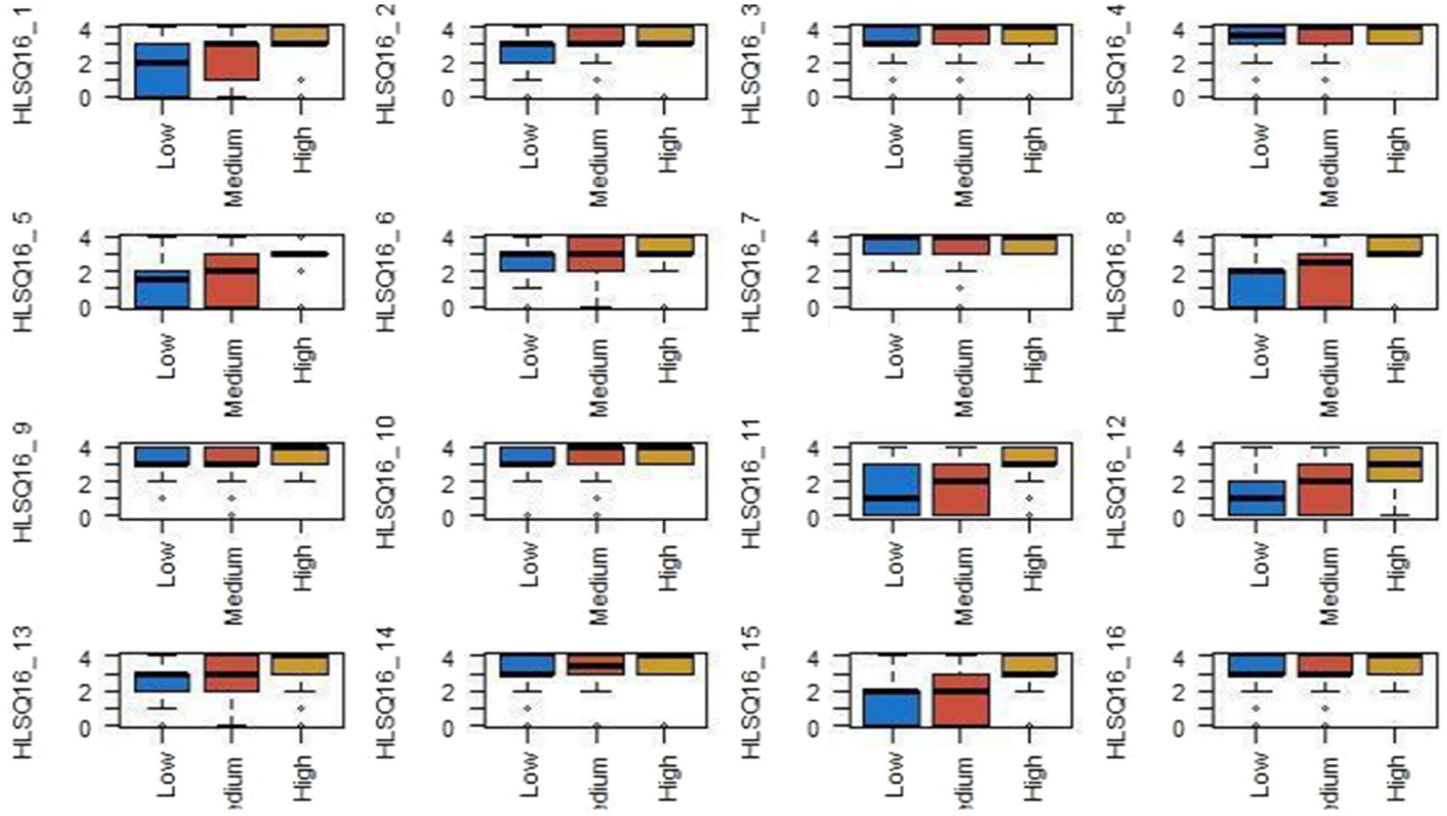
Social class and health literacy.

Analysis of HL scores according to social class, using the Kruskal–Wallis nonparametric test, reflects statistically significant differences in several items of the HLS-Q16 scale. The most affected items were those related to the understanding and application of health information, specifically, items HLS-Q16_4 (*p* = 0.013), HLS-Q16_5 (*p* = 0.040), HLS-Q16_6 (*p* = 0.001), HLS-Q16_7 (*p* = 0.009), HLS-Q16_10 (*p* = 0.025), HLS-Q16_11 (*p* = 0.018), HLS-Q16_12 (*p* = 0.008), HLS-Q16_13 (*p* = 0.039), HLS-Q16_14 (*p* = 0.012), and HLS-Q16_15 (*p* = 0.004) (Figure 3).

Multiple linear regression models were performed to analyze the joint effect of age, sex, educational level, and social class on the HLS-Q16 scale. College education and being female were consistently associated with higher scores, whereas age showed negative effects on some items. The model indicates that the mean value of the total_HLSQ16, when the independent variables are zero, is 37.799 (*p* < 0.001; CI: 34.455 to 41.143). Furthermore, using the model with total score as the dependent variable (*R*^2^ = 0.179), university education presented the largest positive effect (*B* = 14.006, *p* < 0.001), followed by basic education (*B* = 4.961, *p* = 0.009) and female gender (*B* = 4.863, *p* = 0.010) (Table 3).

**Table 3:** Relationship between social determinants and HL level.

Item	Significant predictors	*β* (beta)	*p*-valor
HLS-Q16_8	Age (−), University education (+)	−0.153/+0.406	0.030/<0.001
HLS-Q16_11	Age (−), Female (+), University education (+)	−0.147/+0.158/+0.250	0.038/0.021/<0.001
HLS-Q16_12	Female (+), Basic education (+), University education (+)	+0.217/+0.200/+0.345	0.002/0.021/<0.001
HLS-Q16_13	Female (+), University education (+)	+0.240/+0.210	<0.001/0.003
HLS-Q16_15	Female (+), University education (+)	+0.182/+0.228	0.009/0.002
HLS-Q16_16	Female (+), Basic education (+), University education (+)	+0.220/+0.181/+0.255	0.002/0.040/0.004

Regression model.

The results of the multiple linear regression for each of the literacy questionnaire scores can be seen in Table 4.

**Table 4:** Relationship between social determinants and each of the literacy questionnaire question.

		Non-standardized coefficients		Standardized coefficients	*t*	Sig.	95.0% CI for *B*	
		*B*	Desv. error	Beta			Lower limit	Upper limit
HLS-Q16_1	(Constant)	2.589	0.093		27.719	<0.001	2.404	2.773
	Upper class	−1.255	0.753	−0.119	−1.667	0.097	−2.740	0.230
HLS-Q16_3	(Constant)	2.306	0.199		11.567	<0.001	1.912	2.699
	Basic education	−0.488	0.226	−0.192	−2.160	0.032	−0.934	−0.042
	University education	−0.851	0.288	−0.263	−2.953	0.004	−1.420	−0.283
HLS-Q16_4	(Constant)	3.377	0.062		54.648	<0.001	3.255	3.498
	University education	0.290	0.150	0.138	1.932	0.055	−0.006	0.586
HLS-Q16_5	(Constant)	1.306	0.219		5.961	<0.001	0.874	1.738
	Basic education	0.575	0.248	0.198	2.317	0.022	0.086	1.065
	University education	1.604	0.317	0.432	5.063	<0.001	0.979	2.228
HLS-Q16_6	(Constant)	2.298	0.248		9.271	<0.001	1.809	2.787
	Middle class	0.460	0.262	0.132	1.754	0.081	−0.057	0.977
	Upper class	1.329	0.728	0.143	1.827	0.069	−0.106	2.764
	University education	0.373	0.225	0.122	1.656	0.099	−0.071	0.817
HLS-Q16_7	(Constant)	3.148	0.150		20.931	<0.001	2.852	3.445
	Middle class	0.307	0.158	0.138	1.948	0.053	−0.004	0.619
	University education	0.288	0.138	0.147	2.081	0.039	0.015	0.560
HLS-Q16_8	(Constant)	3.259	0.857		3.804	<0.001	1.569	4.949
	Age	−0.025	0.011	−0.153	−2.191	0.030	−0.047	−0.002
	Basic education	0.490	0.255	0.163	1.918	0.057	−0.014	0.993
	University education	1.553	0.338	0.406	4.594	<0.001	0.886	2.219
HLS-Q16_9	(Constant)	3.204	0.072		44.214	<0.001	3.061	3.347
	University education	0.402	0.176	0.162	2.284	0.023	0.055	0.750
HLS-Q16_10	(Constant)	2.965	0.162		18.342	<0.001	2.646	3.284
	Middle class	0.466	0.170	0.192	2.748	0.007	0.131	0.800
	University education	0.367	0.149	0.172	2.468	0.014	0.074	0.660
HLS-Q16_11	(Constant)	3.311	0.824		4.020	<0.001	1.686	4.936
	Age	−0.024	0.012	−0.147	−2.087	0.038	−0.047	−0.001
	Female	0.616	0.266	0.158	2.319	0.021	0.092	1.140
	University education	0.975	0.275	0.250	3.539	<0.001	0.432	1.518
HLS-Q16_12	(Constant)	1.198	0.236		5.078	<0.001	0.732	1.663
	Female	0.841	0.264	0.217	3.183	0.002	0.320	1.362
	Basic education	0.608	0.261	0.200	2.329	0.021	0.093	1.123
	University education	1.337	0.333	0.345	4.010	<0.001	0.679	1.994
HLS-Q16_13	(Constant)	2.436	0.108		22.642	<0.001	2.224	2.649
	Female	0.838	0.240	0.240	3.490	<0.001	0.364	1.312
	University education	0.735	0.240	0.210	3.059	0.003	0.261	1.208
HLS-Q16_14	(Constant)	2.762	0.247		11.183	<0.001	2.275	3.249
	Middle class	0.466	0.262	0.135	1.781	0.076	−0.050	0.982
	Upper class	1.238	0.699	0.134	1.772	0.078	−0.140	2.616
HLS-Q16_15	(Constant)	1.276	0.306		4.177	<0.001	0.674	1.879
	Female	0.709	0.267	0.182	2.658	0.009	0.183	1.235
	Middle class	0.578	0.323	0.130	1.791	0.075	−0.059	1.215
	Upper class	1.501	0.894	0.126	1.679	0.095	−0.262	3.264
	University education	0.890	0.277	0.228	3.211	0.002	0.343	1.436
HLS-Q16_16	(Constant)	2.870	0.148		19.346	<0.001	2.577	3.162
	Female	0.526	0.166	0.220	3.167	0.002	0.199	0.854
	Basic education	0.340	0.164	0.181	2.070	0.040	0.016	0.664
	University education	0.612	0.210	0.255	2.917	0.004	0.198	1.025

### 3.2 Results related to self-care

When analyzing the relationship between HL (HLS-EU-Q16) and self-care (EHFScBS), we found a negative and significant overall correlation (*r* = −0.320; *p* < 0.001), suggesting that higher HL was associated with better self-care. Several dimensions of self-care, such as EHFScBS 3 and 8, showed negative correlations with different HLS-EU-Q16 items, especially those related to accessing and understanding information (e.g., HLS-Q16_2, HLS-Q16_3, HLS-Q16_4, and HLS-Q16_5). Item HLS-Q16_9 presented the highest number of significant associations (Table 5).

**Table 5:** Relationship between self-care and HL.

		EHFSCBS 1	EHFSCBS 2	EHFSCBS 3	EHFSCBS 4	EHFSCBS 5	EHFSCBS 6	EHFSCBS 7	EHFSCBS 8	EHFSCBS 9	EHFSCBS 10	EHFSCBS 11	EHFSCBS 12
HLS-Q16_1	Correlation coefficient	0.008	−0.087	−0.187^**^	−0.073	−0.106	−0.046	0.062	−0.158^*^	−0.026	−0.090	−0.002	−0.024
	Sig. (bilateral)	0.907	0.227	0.009	0.308	0.140	0.524	0.389	0.028	0.714	0.210	0.976	0.738
HLS-Q16_2	Correlation coefficient	0.105	−0.159^*^	−0.079	−0.110	0.020	−0.131	−0.071	−0.172^*^	−0.011	−0.187^**^	−0.056	−0.083
	Sig. (bilateral)	0.144	0.027	0.270	0.125	0.781	0.068	0.326	0.016	0.883	0.009	0.437	0.249
HLS-Q16_3	Correlation coefficient	−0.013	−0.117	−0.169^*^	−0.097	−0.096	−0.263^**^	−0.047	−0.100	−0.014	−0.225^**^	−0.139	0.039
	Sig. (bilateral)	0.854	0.105	0.018	0.179	0.184	<0.001	0.515	0.167	0.845	0.002	0.052	0.594
HLS-Q16_4	Correlation coefficient	0.039	−0.187^**^	−0.170^*^	−0.111	−0.104	−0.192^**^	−0.050	−0.148^*^	0.049	−0.216^**^	−0.153^*^	−0.029
	Sig. (bilateral)	0.588	0.009	0.018	0.123	0.148	0.007	0.487	0.039	0.498	0.002	0.032	0.691
HLS-Q16_5	Correlation coefficient	−0.096	−0.186^**^	−0.300^**^	−0.194^**^	−0.114	−0.072	0.051	−0.251^**^	−0.104	−0.023	0.043	−0.048
	Sig. (bilateral)	0.182	0.009	<0.001	0.007	0.112	0.317	0.480	<0.001	0.148	0.751	0.552	0.506
HLS-Q16_6	Correlation coefficient	−0.050	−0.053	−0.244^**^	−0.137	−0.181^*^	−0.081	0.077	−0.185^**^	0.085	−0.110	−0.064	−0.097
	Sig. (bilateral)	0.489	0.465	<0.001	0.056	0.011	0.259	0.284	0.010	0.239	0.126	0.375	0.176
HLS-Q16_7	Correlation coefficient	0.034	−0.165^*^	−0.225^**^	−0.147^*^	−0.163^*^	−0.169^*^	−0.080	−0.191^**^	0.029	−0.252^**^	−0.143^*^	−0.075
	Sig. (bilateral)	0.636	0.021	0.002	0.040	0.023	0.018	0.266	0.007	0.685	<0.001	0.046	0.296
HLS-Q16_8	Correlation coefficient	−0.099	−0.143^*^	−0.179^*^	−0.143^*^	−0.103	−0.059	−0.060	−0.245^**^	−0.010	−0.028	−0.042	0.007
	Sig. (bilateral)	0.169	0.046	0.012	0.046	0.150	0.410	0.403	<0.001	0.889	0.699	0.561	0.918
HLS-Q16_9	Correlation coefficient	−0.069	−0.261^**^	−0.178^*^	−0.221^**^	−0.270^**^	−0.145^*^	−0.029	−0.168^*^	−0.162^*^	−0.178^*^	−0.184^*^	−0.147^*^
	Sig. (bilateral)	0.335	<0.001	0.013	0.002	<0.001	0.044	0.690	0.019	0.023	0.013	0.010	0.041
HLS-Q16_10	Correlation coefficient	0.032	−0.242^**^	−0.297^**^	−0.234^**^	−0.161^*^	−0.096	−0.005	−0.245^**^	−0.032	−0.246^**^	−0.183^*^	−0.003
	Sig. (bilateral)	0.653	<0.001	<0.001	<0.001	0.025	0.181	0.944	<0.001	0.654	<0.001	0.011	0.966
HLS-Q16_11	Correlation coefficient	−0.022	−0.190^**^	−0.167^*^	−0.102	−0.126	−0.157^*^	−0.025	−0.233^**^	−0.068	0.006	−0.002	−0.009
	Sig. (bilateral)	0.763	0.008	0.020	0.157	0.081	0.028	0.724	0.001	0.348	0.934	0.983	0.905
HLS-Q16_12	Correlation coefficient	−0.049	−0.195^**^	−0.122	−0.021	−0.077	−0.224^**^	−0.057	−0.190^**^	−0.133	−0.024	0.041	−0.076
	Sig. (bilateral)	0.498	0.006	0.090	0.771	0.285	0.002	0.430	0.008	0.065	0.740	0.567	0.291
HLS-Q16_13	Correlation coefficient	−0.027	−0.201^**^	−0.175^*^	−0.153^*^	−0.143^*^	−0.196^**^	−0.122	−0.311^**^	−0.075	−0.056	0.049	−0.288^**^
	Sig. (bilateral)	0.710	0.005	0.015	0.032	0.045	0.006	0.090	<0.001	0.299	0.438	0.495	<0.001
HLS-Q16_14	Correlation coefficient	−0.034	−0.152^*^	−0.247^**^	−0.257^**^	−0.178^*^	−0.139	0.052	−0.278^**^	−0.019	−0.169^*^	−0.052	−0.068
	Sig. (bilateral)	0.635	0.034	<0.001	<0.001	0.013	0.053	0.472	<0.001	0.791	0.018	0.469	0.346
HLS-Q16_15	Correlation coefficient	−0.083	−0.227^**^	−0.137	−0.089	−0.125	−0.168^*^	−0.050	−0.190^**^	−0.038	−0.024	0.025	−0.077
	Sig. (bilateral)	0.251	0.001	0.055	0.216	0.081	0.019	0.489	0.008	0.603	0.734	0.726	0.284
HLS-Q16_16	Correlation coefficient	−0.003	−0.092	−0.094	−0.077	−0.055	−0.160^*^	−0.121	−0.118	−0.095	−0.176^*^	−0.063	−0.153^*^
	Sig. (bilateral)	0.969	0.199	0.193	0.286	0.449	0.025	0.091	0.100	0.188	0.014	0.378	0.033

Higher EHFScB-9 scores denote poorer self-care; lower scores denote better self-care. The ** is the statistical significance. There is statistical evidence of a negative (inverse) relationship between these variables.

A multivariable linear regression model was used to analyze self-care (EHFScBS) as a function of HL (HLS-EU-Q16) and sociodemographic variables. HL remained independently associated with lower EHFScB-9 scores (*B* = −0.189; *p* < 0.001) confirming this relationship. Likewise, belonging to the upper-middle social class was associated with a lower self-care score (*B* = −5.040; *p* = 0.001), while female sex was associated with a significant increase in self-care score (*B* = 2.957; *p* = 0.018), controlling for the other variables in the model (Table 6).

**Table 6:** Relationship between social determinants, self-care and HL.

Model		Non-standardized coefficients		Standardized coefficients	*t*	Sig.	95% CI for *B*	
		*B*	Desv. error	Beta			Lower limit	Upper limit
3	(Constant)	34.020	2.221		15.314	<0.001	29.638	38.402
	TOTAL_HLSQ16	−0.189	0.045	−0.291	−4.257	<0.001	−0.277	−0.102
	Upper-middle class	−5.040	1.546	−0.221	−3.259	0.001	−8.090	−1.990
	Gender female	2.957	1.243	0.160	2.380	0.018	0.506	5.409

Finally, analysis of the relationship between health literacy (HLS-EU-Q16) and self-care ability (EHFScBS) showed an overall correlation of −0.320 (*p* < 0.001). Most of the significant correlations were negative, suggesting that higher levels of health literacy might be associated with lower scores on the EHFScBS scale, and indicating that higher levels of health literacy might be associated with better self-care (Table 7).

**Table 7:** Relationship between self-care ability and HL level.

		EHFSCBS 1	EHFSCBS 2	EHFSCBS 3	EHFSCBS 4	EHFSCBS 5	EHFSCBS 6	EHFSCBS 7	EHFSCBS 8	EHFSCBS 9	EHFSCBS 10	EHFSCBS 11	EHFSCBS 12
HLS-Q16_1	Correlation coefficient	0.008	−0.087	−0.187^**^	−0.073	−0.106	−0.046	0.062	−0.158^*^	−0.026	−0.090	−0.002	−0.024
	Sig. (bilateral)	0.907	0.227	0.009	0.308	0.140	0.524	0.389	0.028	0.714	0.210	0.976	0.738
HLS-Q16_2	Correlation coefficient	0.105	−0.159^*^	−0.079	−0.110	0.020	−0.131	−0.071	−0.172^*^	−0.011	−0.187^**^	−0.056	−0.083
	Sig. (bilateral)	0.144	0.027	0.270	0.125	0.781	0.068	0.326	0.016	0.883	0.009	0.437	0.249
HLS-Q16_3	Correlation coefficient	−0.013	−0.117	−0.169^*^	−0.097	−0.096	−0.263^**^	−0.047	−0.100	−0.014	−0.225^**^	−0.139	0.039
	Sig. (bilateral)	0.854	0.105	0.018	0.179	0.184	<0.001	0.515	0.167	0.845	0.002	0.052	0.594
HLS-Q16_4	Correlation coefficient	0.039	−0.187^**^	−0.170^*^	−0.111	−0.104	−0.192^**^	−0.050	−0.148^*^	0.049	−0.216^**^	−0.153^*^	−0.029
	Sig. (bilateral)	0.588	0.009	0.018	0.123	0.148	0.007	0.487	0.039	0.498	0.002	0.032	0.691
HLS-Q16_5	Correlation coefficient	−0.096	−0.186^**^	−0.300^**^	−0.194^**^	−0.114	−0.072	0.051	−0.251^**^	−0.104	−0.023	0.043	−0.048
	Sig. (bilateral)	0.182	0.009	<0.001	0.007	0.112	0.317	0.480	<0.001	0.148	0.751	0.552	0.506
HLS-Q16_6	Correlation coefficient	−0.050	−0.053	−0.244^**^	−0.137	−0.181^*^	−0.081	0.077	−0.185^**^	0.085	−0.110	−0.064	−0.097
	Sig. (bilateral)	0.489	0.465	<0.001	0.056	0.011	0.259	0.284	0.010	0.239	0.126	0.375	0.176
HLS-Q16_7	Correlation coefficient	0.034	−0.165^*^	−0.225^**^	−0.147^*^	−0.163^*^	−0.169^*^	−0.080	−0.191^**^	0.029	−0.252^**^	−0.143^*^	−0.075
	Sig. (bilateral)	0.636	0.021	0.002	0.040	0.023	0.018	0.266	0.007	0.685	<0.001	0.046	0.296
HLS-Q16_8	Correlation coefficient	−0.099	−0.143^*^	−0.179^*^	−0.143^*^	−0.103	−0.059	−0.060	−0.245^**^	−0.010	−0.028	−0.042	0.007
	Sig. (bilateral)	0.169	0.046	0.012	0.046	0.150	0.410	0.403	<0.001	0.889	0.699	0.561	0.918
HLS-Q16_9	Correlation coefficient	−0.069	−0.261^**^	−0.178^*^	−0.221^**^	−0.270^**^	−0.145^*^	−0.029	−0.168^*^	−0.162^*^	−0.178^*^	−0.184^*^	−0.147^*^
	Sig. (bilateral)	0.335	<0.001	0.013	0.002	<0.001	0.044	0.690	0.019	0.023	0.013	0.010	0.041
HLS-Q16_10	Correlation coefficient	0.032	−0.242^**^	−0.297^**^	−0.234^**^	−0.161^*^	−0.096	−0.005	−0.245^**^	−0.032	−0.246^**^	−0.183^*^	−0.003
	Sig. (bilateral)	0.653	<0.001	<0.001	<0.001	0.025	0.181	0.944	<0.001	0.654	<0.001	0.011	0.966
HLS-Q16_11	Correlation coefficient	−0.022	−0.190^**^	−0.167^*^	−0.102	−0.126	−0.157^*^	−0.025	−0.233^**^	−0.068	0.006	−0.002	−0.009
	Sig. (bilateral)	0.763	0.008	0.020	0.157	0.081	0.028	0.724	0.001	0.348	0.934	0.983	0.905
HLS-Q16_12	Correlation coefficient	−0.049	−0.195^**^	−0.122	−0.021	−0.077	−0.224^**^	−0.057	−0.190^**^	−0.133	−0.024	0.041	−0.076
	Sig. (bilateral)	0.498	0.006	0.090	0.771	0.285	0.002	0.430	0.008	0.065	0.740	0.567	0.291
HLS-Q16_13	Correlation coefficient	−0.027	−0.201^**^	−0.175^*^	−0.153^*^	−0.143^*^	−0.196^**^	−0.122	−0.311^**^	−0.075	−0.056	0.049	−0.288^**^
	Sig. (bilateral)	0.710	0.005	0.015	0.032	0.045	0.006	0.090	<0.001	0.299	0.438	0.495	<0.001
HLS-Q16_14	Correlation coefficient	−0.034	−0.152^*^	−0.247^**^	−0.257^**^	−0.178^*^	−0.139	0.052	−0.278^**^	−0.019	−0.169^*^	−0.052	−0.068
	Sig. (bilateral)	0.635	0.034	<0.001	<0.001	0.013	0.053	0.472	<0.001	0.791	0.018	0.469	0.346
HLS-Q16_15	Correlation coefficient	−0.083	−0.227^**^	−0.137	−0.089	−0.125	−0.168^*^	−0.050	−0.190^**^	−0.038	−0.024	0.025	−0.077
	Sig. (bilateral)	0.251	0.001	0.055	0.216	0.081	0.019	0.489	0.008	0.603	0.734	0.726	0.284
HLS-Q16_16	Correlation coefficient	−0.003	−0.092	−0.094	−0.077	−0.055	−0.160^*^	−0.121	−0.118	−0.095	−0.176^*^	−0.063	−0.153^*^
	Sig. (bilateral)	0.969	0.199	0.193	0.286	0.449	0.025	0.091	0.100	0.188	0.014	0.378	0.033

Higher EHFScB-9 scores denote poorer self-care; lower scores denote better self-care. The ** is the statistical significance. There is statistical evidence of a negative (inverse) relationship between these variables.

Linear regression between HLSQ16 and EHFScBS total scores indicates that, in the absence of other factors, the model estimates a mean self-care score of 34.02. Furthermore, for each additional point in health literacy (HLSQ16), the self-care score (EHFScBS) is reduced by an average of 0.189 points (*p* < 0.001), again indicating a relationship between higher levels of HL and better self-care (Table 8).

**Table 8:** Relationship between self-care scores and HL.

Model		Non-standardized coefficients		Standardized coefficients	*t*	Sig.	95 CI for *B*	
		*B*	Desv. error	Beta			Lower limit	Upper limit
3	(Constant)	34.02	2.221		15.314	<0.001	29.638	38.402
	TOTAL_HLSQ16	−0.189	0.045	−0.291	−4.257	<0.001	−0.277	−0.102
	Upper-middle class	−5.04	1.546	−0.221	−3.259	0.001	−8.09	−1.99
	Gender female	2.957	1.243	0.16	2.38	0.018	0.506	5.409

## 4 Discussion

Our study analyzed health literacy (HL) and self-care in patients with heart failure (HF) in a region of Spain using validated questionnaires (HLS-EU-Q16 and EHFScBS, respectively). Sociodemographic factors such as sex, educational level, or perceived social class, influence different dimensions of HL, whereas age and educational level influence self-care.

As with other health problems, HL is essential for maintaining the health of people with HF (31). HL enables effective communication with healthcare professionals, appropriate treatment follow-up, and informed decision-making about self-care, while also fostering critical and social skills that empower patients in their daily lives and healthcare environments (32). Conversely, low HL is associated with poorer medication adherence, greater use of emergency services, worse disease control, and higher risk of hospitalization (27, 33).

In line with the literature, educational level in our participants was positively associated with higher scores across multiple HL dimensions, reinforcing the importance of formal education in promoting health competencies (34). Another factor closely linked to education and likewise associated with better HL was perceived social class, which reflects a confluence of structural factors (e.g., economic resources and opportunities for personal development) (35).

In our study, participants at upper-middle social class had lower self-care. This counterintuitive finding could be explained by behaviors related to the delegation of care to other people, more stressful lifestyles, lower risk perception, or greater confidence in healthcare interventions versus self-care. Poor self-care behaviors have been previously described in populations with higher income or at higher socioeconomic position in highly prevalent chronic conditions such as diabetes (36, 37). Specifically, Walker et al. (37) found that higher household income (>$75,000) was associated with poorer self-care regarding exercise, in terms of fewer days per week of physical activity and lower performance. By contrast, in Burch et al.’s study (36), individuals at higher socioeconomic position performed more exercise and consumed more vegetables; however, they also exhibited worse self-care behaviors than individuals in lower social position regarding self-monitoring of blood glucose, foot inspection and care (prevention of diabetic foot), and treatment adherence. Thus, we argue against individualistic perspectives of HL and support the importance of the socioeconomic context of individuals (18, 32).

Regarding self-care, our older and less educated participants had higher scores on the EHFScBS scale, or lower level of self-care. These findings suggest a weak, but significant, relationship between older age and specific components/dimensions of HL in certain specific aspects. These results coincide with previous studies focused on self-care in heart failure (38). In Cajita et al. (39), patients with better HL were more competent in monitoring and managing their HF. In our study, however, the HL-HF association was positive with self-care maintenance. In previous studies, patients with higher HL might follow better maintenance activities, such as medication adherence and symptom monitoring, but might not be as proactive in managing acute symptoms (40). These findings could reflect the complexity and multi-factoriality of the relationship between HL and self-care, including the relative contribution of factors already mentioned such as age, educational level, social support, or others such as comorbidities (41), perceived self-efficacy, or intrinsic motivation (40, 42).

Beyond individual abilities, the social component—through mutual support and coping styles—indirectly shapes symptom burden and remains an undervalued, underexploited lever in therapeutic interventions (43). Consistent with this, HL in people with CHF may influence symptom burden indirectly via social support and coping, with a need to design interventions tailored to each person’s capabilities and context (27, 33, 43). Moreover, the association between HL and self-care obtained in our study highlights the importance of integrating interventions in both areas to foster optimal self-care behaviors.

With respect to sex, our results show that, compared with men, women reported significantly higher scores on several items of the HLS scale. This finding could be related to sociocultural, gender role and behavioral factors in accessing and using health information (44), and suggesting greater female involvement in health care and information seeking related to health (13, 45). This pattern supports the need to incorporate a gender perspective when designing educational interventions to maximize effectiveness.

Accordingly, the most successful educational interventions in people with HF have been those personalized (46), such as structured and individualized educational programs that have significantly improved knowledge of the disease and therapeutic adherence. Clear guidance would still be lacking to address this synergy of HF promotion activities with self-care, with interventions that are not only effective but also scalable scientific (31), as well as assessing the sustainability of long-term effects and their impact on clinical outcomes such as hospitalizations and mortality (31, 42, 47).

Regarding clinical variables, no significant differences in HL or self-care were found between patients with or without hypertension, dyslipidemia or smoking. However, specific differences in self-care items were identified in people with diabetes, obesity and dyslipidemia, suggesting a possible influence of certain comorbidities. The frequency of hospitalizations and consultations was not consistently associated with HL, although some items showed point correlations, possibly linked to the perception of symptoms or need for care (42). Finally, although the NYHA classification did not show global differences in the scales, certain self-care items did correlate with clinical stage, which could reflect behavioral adaptation rather than differences in HL.

A secondary objective in our study was to explore the predictive capacity of HL screening on self-care capacity in HF. In this respect, the results appear conclusive: low HL scores may result in poorer HF management ability. Jiménez-Méndez et al. (48) argue that good predictive validity—the ability of a tool to accurately predict future outcomes—would help identify patients at high risk of decompensated HF, which, as shown, often results from self-care deficits. Identifying these at-risk patients will facilitate the implementation of personalized preventive strategies.

### 4.1 Implications for practice

The results encourage the development of specific strategies to reduce social inequalities in HL. From a nursing perspective, these findings further highlight the importance of assessing patients’ HL as an integral part of care, using validated tools such as the HLS-EU-Q16, which as seen, would allow prediction of self-care capacity in HF. It is vital to incorporate a gender perspective, recognizing the challenges that affect men and women in the management of their health, and to acknowledge the weight of the intersectionality of these characteristics. It is still necessary to develop support programs focused not only on women (or men) but on those segments more vulnerable by education or socioeconomic position, with a holistic view, as well as to obtain evidence on HL and self-care in traditionally neglected populations. These interventions should not only be clinical but focused on mitigating the effects of these determinants on HL and thus, self-care, through public policies that recognize HL as a key social mediator, favoring justice, equity, and efficiency of the health system.

Future research should address these limitations by designing longitudinal studies that allow us to observe the evolution of HL and self-care over time, as well as their influence on relevant clinical outcomes such as re-hospitalizations or quality of life. It would be useful to implement and evaluate personalized educational interventions according to the level of HL, incorporating adapted communication strategies and digital tools. It is also proposed to replicate the study in other geographical settings and with more diverse samples to improve external validity. The incorporation of qualitative approaches would allow us to explore in greater depth the barriers and facilitators perceived by patients in their interaction with the healthcare system. Finally, it would be valuable to use instruments that assess not only health information comprehension, but also the critical and functional skills that enable autonomous and effective health management.

### 4.2 Limitations

Our study presents several limitations to consider when interpreting the results. First, its cross-sectional design precludes establishing causal relationships between HL and self-care behaviors. In addition, the HLS-EU-Q16 questionnaire, although useful and validated, only partially captures the complexity of HL, focusing on stated dimensions rather than actual functional abilities. It is also important to note the potential bias introduced by the high proportion of male participants (83.1%), which together with recruitment from a single center, may affect the generalizability of results to other populations or healthcare settings. It is likewise possible that the use of telephone interviews and the self-reported nature of the questionnaires may have introduced response bias or social desirability. Finally, although relevant clinical variables were collected, their analysis was not central to this work, so their relationship with HL and self-care was underexplored.

## 5 Conclusion

HL in people with HF is associated with factors such as sex, educational level, and socioeconomic position. However, these findings underline that, although clinical research provides valuable data, sustained improvement of HL and self-care in HF requires structural social interventions, beyond the clinical setting. There is a need to move toward approaches that integrate public policies, tailored educational strategies, and cross-sectoral actions that address the structural inequalities that underlie differences in HL. Increasing evidence is useful, but the real impact will come from social interventions that promote critical, equitable, and contextualized literacy.

## Data Availability

The raw data supporting the conclusions of this article will be made available by the authors, without undue reservation.
